# A systematic review and meta-analysis including GRADE qualification of the risk of surgical site infections after prophylactic negative pressure wound therapy compared with conventional dressings in clean and contaminated surgery

**DOI:** 10.1097/MD.0000000000004673

**Published:** 2016-09-09

**Authors:** Fleur E.E. De Vries, Elon D. Wallert, Joseph S. Solomkin, Benedetta Allegranzi, Matthias Egger, E. Patchen Dellinger, Marja A. Boermeester

**Affiliations:** a Department of Surgery, Academic Medical Centre Amsterdam, The Netherlands; b Department of Surgery, University of Cincinnati College of Medicine, Cincinnati, OH, USA; c Infection Prevention and Control Global Unit, Service Delivery and Safety, World Health Organization, Geneva, Switzerland; d Institute of Social and Preventive medicine, University of Bern, Switzerland; e Department of Surgery, University of Washington, Seattle, WA, USA.

**Keywords:** incisional wound therapy, prevention, prophylactic negative pressure wound therapy, surgical site infection, wound infections

## Abstract

**Objective:**

Systematically review and Grading of Recommendations Assessment, Development, and Evaluation (GRADE) studies on prophylactic negative pressure wound therapy (pNPWT) to prevent surgical site infections (SSIs).

**Introduction:**

pNPWT has been suggested as a new method to prevent wound complications, specifically SSIs, by its application on closed incisional wounds.

**Methods:**

This review was conducted as part of the development of the Global Guidelines for prevention of SSIs commissioned by World Health Organization in Geneva. PubMed, Embase, CENTRAL, CINAHL, and the World Health Organization database between January 1, 1990 and October 7, 2015 were searched. Inclusion criteria were randomized controlled trials and observational studies comparing pNPWT with conventional wound dressings and reporting on the incidence of SSI. Meta-analyses were performed with a random effect model. GRADE Pro software was used to qualify the evidence.

**Results:**

Nineteen articles describing 21 studies (6 randomized controlled trials and 15 observational) were included in the review. Summary estimate showed a significant benefit of pNPWT over conventional wound dressings in reducing SSIs in both randomized controlled trials and observational studies, odds ratio of 0.56 (95% confidence interval, 0.32–0.96; *P* = 0.04) and odds ratio of 0.30 (95% confidence interval, 0.22–0.42; *P* < 0.00001), respectively. This translates into lowering the SSI rate from 140 to 83 (49–135) per 1000 patients and from 106 to 34 (25–47) per 1000 patients, respectively. In stratified analyses, these results were consistent in both clean and clean-contaminated procedures and in different types of surgery, however results were no longer significant for orthopaedic/trauma surgery. The level of evidence as qualified with GRADE was however low.

**Conclusions:**

Low-quality evidence indicates that prophylactic NPWT significantly reduces the risk of SSIs.

## Abstract 2

Supplemental Digital Content is available in the text

## 1 Introduction

Surgical site infections (SSIs) are the number one healthcare-associated infections worldwide, with an incidence of 2% to 20%, or even higher, depending on the type of surgery and patient characteristics.^[1,2]^ SSI are associated with increased morbidity, mortality, and extended hospital stay. Furthermore, increased healthcare costs are attributable to SSI.^[3]^

Several perioperative preventive measures have been implemented to minimize the risk of SSI, such as hand washing of the surgical team, antibiotic prophylaxis, skin preparation, and sterile drapes and gowns. Despite these measures healthcare associated infections, especially SSI, remain a challenging problem to surgeons and patients worldwide.^[4]^

Prophylactic (or closed incision) negative pressure wound therapy (pNPWT) denotes the prophylactic use of negative pressure wound therapy (NPWT) to prevent wound complications, specifically SSI. Although NPWT has been used since late 1990s for several purposes, such as open bone fractures,^[5,6]^ diabetic ulcers,^[7]^ and management of the open abdomen,^[8]^ its prophylactic use for primarily closed incisions has only been described a decade ago.^[9]^

Prophylactic NPWT consist of a hermetically sealed system connected to a vacuum pump, which maintains negative pressure on the wound surface. Although several studies on the working mechanism of NPWT have been performed and reviewed,^[10]^ there is a lack of preclinical research regarding pNPWT. It has been suggested that by using negative pressure dead space is reduced, tissue proliferation is stimulated, and fluids are removed. Moreover, pNPWT could protect against microorganisms entering from the outside world. Prophylactic NPWT has been suggested as a promising application to reduce SSIs and other wound complications. A few previous reviews have been published^[11,12]^ using different methodology, for example, combining randomized controlled trials (RCTs) and observational studies into one analysis. Not many studies on clean-contaminated surgery were included, and none of the previous systematic reviews qualified evidence using Grading of Recommendations Assessment, Development, and Evaluation (GRADE). Our aim was to systematically review the available literature on pNPWT in terms of reducing SSI in all types of surgery. This review was conducted as part of the development of the Global Guidelines for prevention of SSIs commissioned by World Health Organization in Geneva.

## 2 Methods

The PRISMA^[13]^ (preferred Reporting Items for Systematic Reviews and Meta-Analyses) and MOOSE^[14]^ (meta-analysis of observational studies in epidemiology) guidelines were followed.

### 2.1 Search strategy and selection criteria

References for this review were identified through searches of PubMed, EMBASE (Ovid), the Cochrane Central Register of Controlled Trials (CENTRAL), CINAHL, and World Health Organization for articles published from January 1990 to October 7, 2015 by use of the terms “surgical site infection,” “negative pressure wound therapy,” and “surgical procedure.” The complete search is included in appendix A. Two authors (FdV and EW) independently screened all titles and abstracts. RCTs or observational studies comparing pNPWT with conventional wound dressings in adult patients were included. We chose to include observational studies, because previous systematic reviews did not identify any RCTs in potentially contaminated surgery. Studies had to, at least, report on SSIs or wound infections, either as primary or secondary outcome. Studies using NPWT on an open wound or split skin graft were excluded. We had no language restrictions. References of the included studies were screened for other relevant studies. We only considered full text published studies.

### 2.2 Data extraction

Study characteristics including year of publication, number of patients, types of surgical procedures, duration of prophylactic NPWT, amount of negative pressure, details on standard dressings, and outcome were retrieved from the text.

### 2.3 Quality assessment

Quality of the included studies was assessed with the Cochrane Collaboration's tool for assessing risk of bias^[15]^ for RCTs and with the New-Castle Ottawa scale^[16]^ for observational studies. The GRADE methodology was used to assess the quality of the body of retrieved evidence (GRADEpro, Version 20. McMaster University, 2014).

### 2.4 Data synthesis and analysis

Meta-analyses were performed with a random effect model in Review Manager (RevMan), Version 5.3. Copenhagen: The Nordic Cochrane Centre, The Cochrane Collaboration, 2014. Heterogeneity of the included studies was evaluated by calculating the *I*^2^ statistic. Subgroup meta-analyses stratified by wound class and type of surgery were performed subsequently.

### 2.5 Ethical approval

Ethical approval was not necessary due to the study design.

## 3 Results

**Figure 1:**
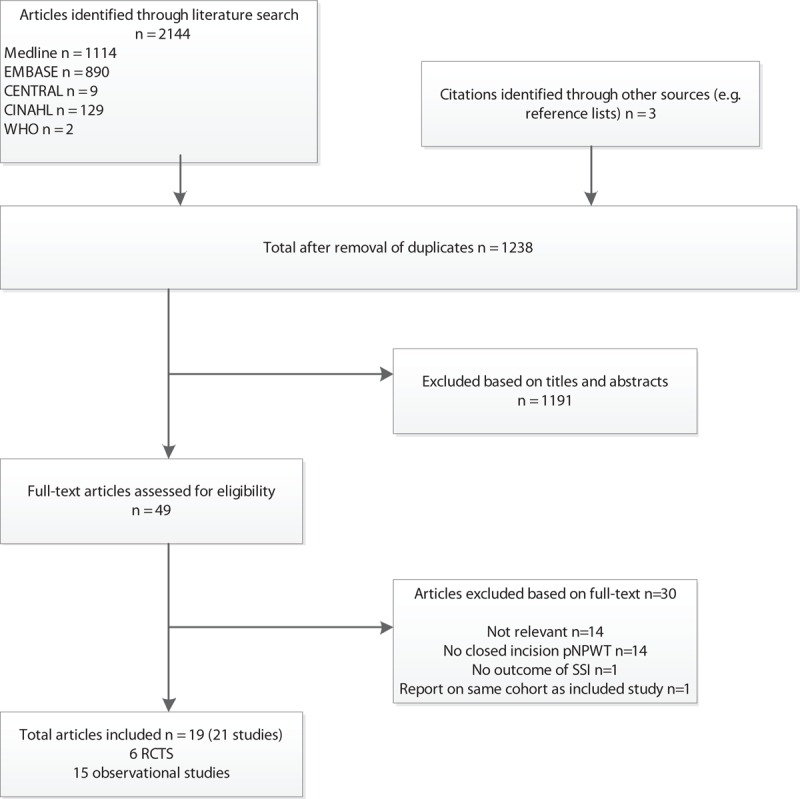
Flow-chart of the systematic review.

Of the 1238 articles initially identified by the search, we selected 49 for a full text review (Fig. 1). Thirty articles were excluded; 14 studies were not relevant, 14 did not describe pNPWT, 1^[17]^ did not report on SSIs as outcome, and 2 studies reported on an overlapping cohort^[18,19]^ and were, therefore, combined for the purpose of analysis. Finally, 19 articles describing 21 studies were included. One article described 2 separate randomized controlled trials within one report,^[9]^ which were assessed and analyzed separately in present meta-analysis. In another observational study, 2 patient populations (breast and colorectal surgery) were assessed and analyzed separately.^[20]^ These were 6 RCTs reported in 5 articles (including 562 patients) and 15 observational studies reported in 14 articles (including 4560 patients).

### 3.1 Study characteristics

We found 6 RCTs,^[6,9,21–23]^ 3 prospective observational studies,^[19,20,24]^ 10 retrospective observational studies,^[25–34]^ and 1 article^[35]^ with both retro- and prospective data. In the observational studies, the use of pNPWT was based on the surgeons decision in 6 studies^[19,25,27,30,32,33]^ and based on time (before-after) in 8 studies.^[24,25,28,29,31,34–36]^ The evidence table with more detailed information is in Table 1.

**Table 1:**
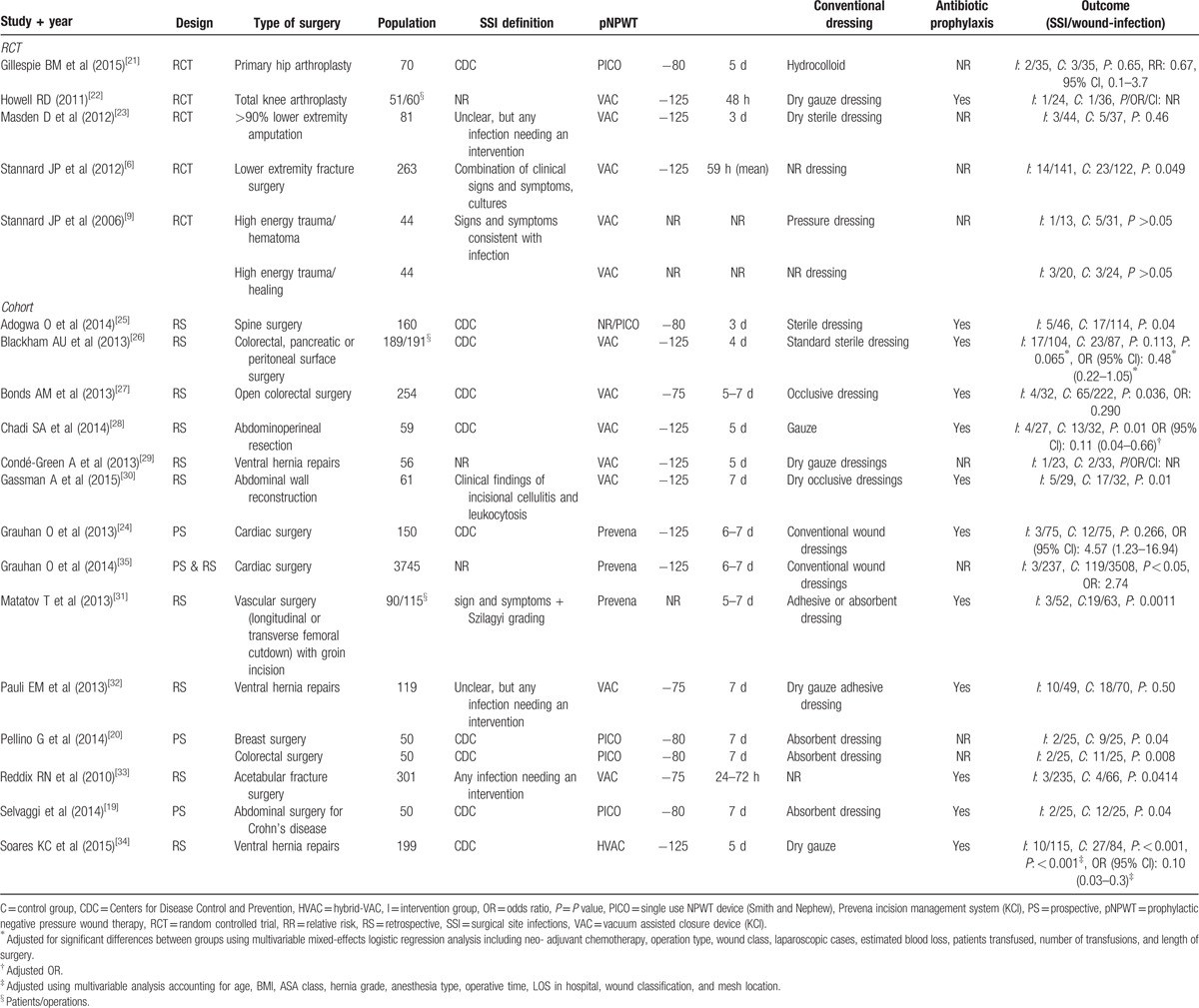
Evidence table.

We found 9 studies on abdominal surgery^[19,26–28,36]^ of which 4 involved ventral hernia repair procedures,^[29,30,32,34]^ 6 studies in orthopaedic or trauma surgery^[9,21,22,25,33]^ 2 studies in cardiac surgery,^[24,35]^ and 2 studies in vascular surgery. One study^[20]^ included both abdominal and breast surgery. Apart from 1 study, all RCTs were performed in clean surgery. The other study involved patients of which more than 90% underwent lower extremity amputation due to chronic wounds. The negative pressure devices were set between 75 and 125 mm Hg, and the length of negative pressure varied between 24 hours till 7 days postoperative. Either dry gauze, conventional-, occlusive-, or absorbent dressings were used in the control group.

The definition for SSI differed between studies. Nine studies used the definition described by the Centers for Disease Control and Prevention,^[37]^ 4 used clinical signs and symptoms as a criteria for SSI, and 2 other, both retrospective studies, scored for SSI in case any treatment was necessary. Follow-up time varied considerably but was, if reported, always at least 30 days. The risk of bias scoring is provided in appendix A.

### 3.2 Prophylactic negative pressure wound therapy versus conventional wound dressings

**Figure 2:**
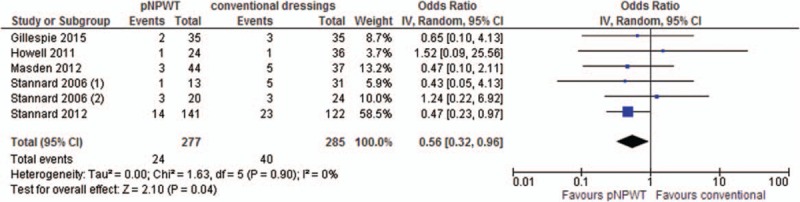
Overall effect of pNPWT on SSI compared to conventional wound dressings in RCTs. pNPWT = prophylactic negative pressure wound therapy, RCT = randomized controlled trial, SSI = surgical site infections.

**Figure 3:**
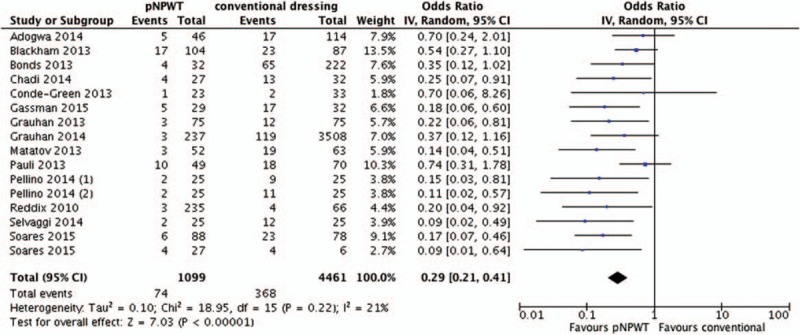
Overall effect of pNPWT on SSI compared to conventional wound dressings in observational studies. pNPWT = prophylactic negative pressure wound therapy, SSI = surgical site infections.

The meta-analysis of 6 RCTs^[6,9,21–23]^ showed a significant benefit of pNPWT over conventional dressings in regard to reducing the risk of SSI (odds ratio [OR], 0.56; 95% confidence interval [CI], 0.32–0.96, *I*^2^ 0%). The meta-analysis of 15 observational studies^[19,20,24–35]^ showed a significant benefit of pNPWT over conventional dressings in regard to reducing the risk of SSI (OR, 0.30; 95% CI, 0.22–0.42, *I*^2^ 18%) (Figs. 2 and 3). The funnel plots of both meta-analyses are included in Appendix B. They did not reveal clear indications of publication bias.

### 3.3 Subgroup comparisons

**Figure 4:**
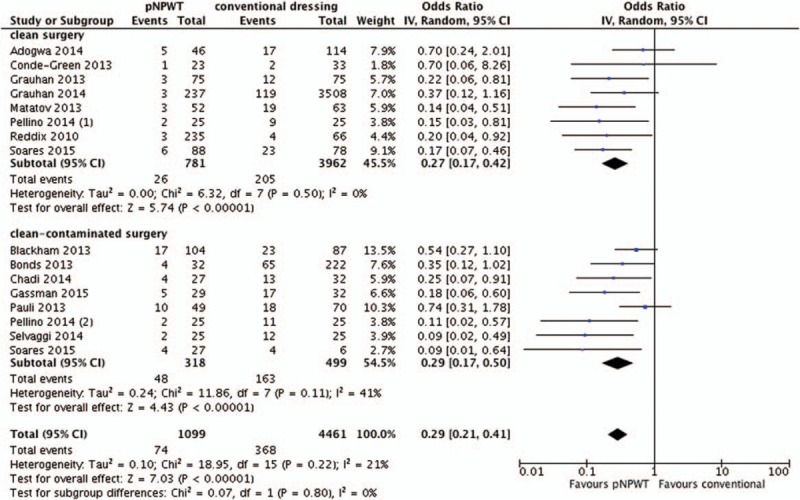
Stratification by wound class (all observational studies).

When we stratified observational studies by wound class the meta-analysis of 8 clean-contaminated or contaminated observational studies^[19,20,26–28,30,32,34]^ showed a significant benefit of pNPWT over conventional dressings (OR, 0.29; 95% CI, 0.17–0.50) as did the meta-analysis of 8 clean observational studies^[19,20,24,25,29,31,33–35]^ (OR, 0.27; 95% CI, 0.17–0.42) (Fig. 4). Two studies^[20,34]^ were used in both aforementioned subgroup comparisons as 1 study reported separate results on both clean and clean-contaminated patients and the other study was included as 2 separate cohort studies in our analysis. When we stratified by type of surgery meta-analysis of 4 RCTs in orthopaedic/trauma surgery did not show significant benefit nor harm in regards to reducing the risk of SSI (OR, 0.58; 95% CI, 0.32–1.07). There was only a single RCT in vascular surgery which did not show a significant reduction in SSI, whereas an observational study in vascular surgery did (OR, 0.47; 95% CI, 0.10–2.11 and OR, 0.14; 95% CI, 0.04–0.51, respectively). The meta-analysis of 9 observational studies in abdominal surgery including, besides the 1 study in vascular surgery, ventral hernia repair showed a significant benefit of pNPWT over conventional dressings in regards to reducing SSI (OR, 0.31; 95% CI, 0.19–0.49) as did 2 studies in cardiac surgery (OR, 0.29; 95% CI, 0.12–0.69), and 1 observational study in breast surgery (OR, 0.15; 95% CI: 0.03–0.81). Two observational studies in orthopaedic/trauma surgery showed no significant benefit for pNPWT over conventional dressings (OR, 0.42; 95% CI, 0.13–1.40). These additional subgroup meta-analyses are included in Appendix C.

### 3.4 GRADE

Overall evidence was qualified using GRADE for both RCTs and observational studies. Overall, low quality of evidence shows that pNPWT may have benefit when compared to conventional postoperative wound dressings in reducing the risk of SSI. The level of evidence for RCTs was downgraded due to the lack of blinding in outcome assessment in most of the studies and because the optimal information size was not met. The GRADE tables are in Tables 2 and 3.

**Table 2:**
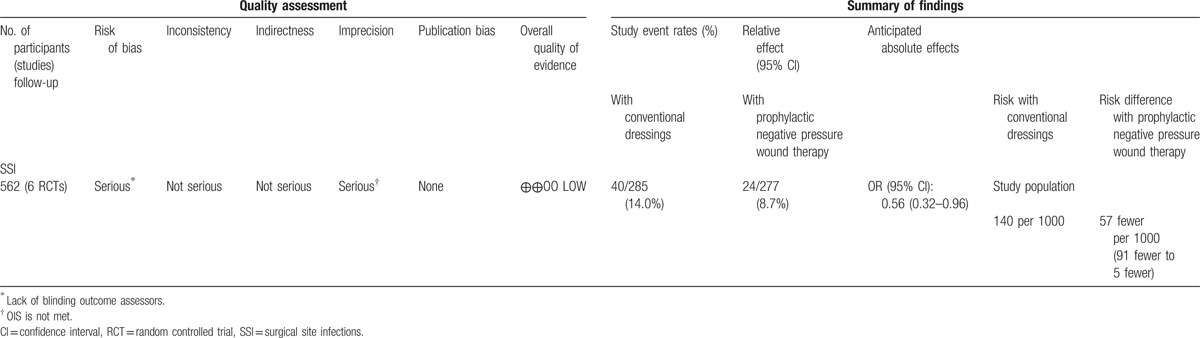
GRADE table RCTs.

**Table 3:**
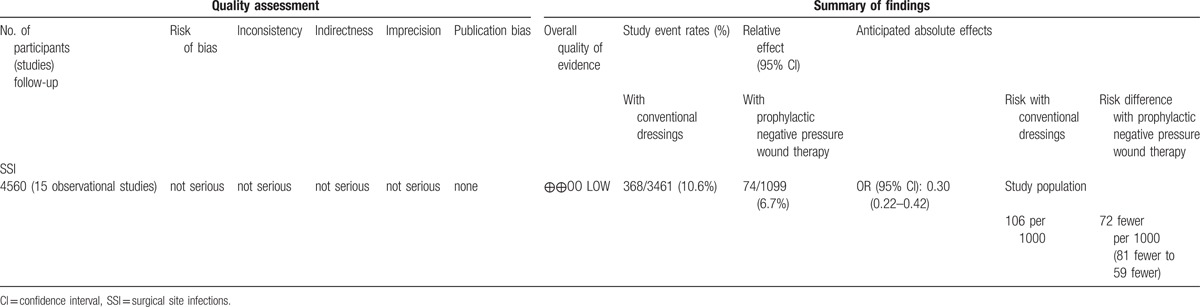
GRADE table observational studies.

## 4 Discussion

In this systematic review, a significant benefit was found for pNPWT over conventional wound dressings in terms of reducing SSIs in both RCTs and observational studies (OR, 0.56; 95% CI, 0.32–0.96 and OR, 0.30; 95% CI, 0.22–0.42, respectively). This translates into lowering the SSI rate from 140 to 83 (49–135) per 1000 patients and from 106 to 34 (25–47) per 1000 patients, respectively. These results were consistent in both clean and clean-contaminated procedures and in different types of surgery. However, in stratified analyses, we found no significant benefit for orthopaedic/trauma surgery in both observational and randomized studies, and conflicting data for vascular surgery. Overall quality of evidence, as qualified by GRADE, was low. This means that further research is very likely to have an important impact on our confidence in the estimate of effect and is likely to change the estimate.^[38]^

Although pNPWT was introduced only 10 years ago, the use of pNPWT has rapidly spread and this application is nowadays used for a variety of wounds. The number of RCTs identified in our literature search is limited and all but 1 RCT were performed following clean, primarily orthopaedic or trauma procedures. We decided to include controlled observational studies because the novelty of the technique is only now generating interest in more accurate RCTs. Moreover, by including observational studies, we included data on potentially contaminated surgery next to clean surgery. In our meta-analyses, we found pNPWT to be more effective in observational studies (OR, 0.30) than RCTs (OR, 0.56). This is expected due to the bias in observational studies where surgeon perceptions, often quite accurate, determine allocation.^[39]^ We note that several observational studies included in our review reported a high incidence of SSI in the control group. Most of these studies were performed in abdominal surgery or (complex) ventral hernia repair, surgical procedures prone to SSI.^[40,41]^ The inherent selection of high risk wounds prevents extrapolating these results to all types of surgery.

This is the first systematic review and meta-analysis performing separate analyses for observational studies and RCTs and qualifying evidence by the use of GRADE. One previous meta-analysis was performed by Semsarzadeh et al,^[12]^ but they combined observational studies and RCTs in their analyses. Additionally, we included 6 recently published studies. Another more recent meta-analysis^[11]^ only included RCTs, but they included unpublished data and studies not reporting on the incidence of SSI but only on seroma.

Some limitations of the present study need to be addressed. In the first place, we used (retrospective) observational studies as well as RCTs. Observational studies are well known to involve selection bias and publication bias, as positive results may be more likely to be published than negative results.^[39]^ Another limitation was the inclusion of only a small number of patients in most studies, and therefore large confidence intervals within each study. This makes it difficult to estimate the real effect. Although statistical heterogeneity was low (*I*^2^ 0% for RCTs and *I*^2^ 18% for observational studies), there was variation in protocols used in the included studies in important variables, such as the amount of negative pressure, the duration of pNPWT, and the control dressing. There was also variation in the definition of a SSI used among the included studies and a lack of baseline characteristics influencing the risk of SSI (eg, diabetes or immunosuppression). Therefore, clinical heterogeneity might be higher than statistical heterogeneity reveals.

Although the potential of pNPWT to reduce the incidence of SSI has been shown, limited studies on cost effectiveness are available. The price of pNPWT varies between $15/d^[42]^ and $495/wk^[43]^ depending on a self-made or commercial application. Three studies on cost effectiveness in a gynecologic population have been performed.^[44–46]^ Their results show a potential for pNPWT to be cost effective, especially in patient groups with high risk of SSI, such as clean-contaminated operations, or operations performed upon patients with intrinsic risk factors, such as obesity, diabetes, or immunosuppressive medication.

This brings us to the question how useful GRADE is when dealing with new technologies, and even more specific, a new application for a relatively new technology. It remains to be seen whether a significant benefit found in meta-analysis but qualified as low (due to high risk of bias and patient selection) is enough to promote its widespread use. On the other hand, surgeons dealing with difficult wounds might want to use every option they have to reduce wound infections.

Conclusive data on cost effectiveness are lacking, but pNPWT is most likely to be cost effective in procedures at high risk for SSI due to patients’ or operative characteristics. Before widespread use of this application in low-risk as well as high-risk wounds, randomized controlled trials are required to identify the group of patients in whom pNPWT is cost effective. Moreover, the influence of pNPWT on other important wound complications, such as wound dehiscence or seroma, has been shown but needs to be studied more specifically in prospective studies.

## Supplementary Material

Supplemental Digital Content
