# Supplementary material for: A systematic review and meta-analysis including GRADE qualification of the risk of surgical site infections after prophylactic negative pressure wound therapy compared with conventional dressings in clean and contaminated surgery
Source: Medicine (Baltimore). 2016 Sep 9;95(36):e4673. doi: 10.1097/MD.0000000000004673 (PMC5023882; doi:10.1097/MD.0000000000004673)

**Appendix A.**

Risk of bias RCTs

| **Author, year** | **Sequence generation** | **Allocation concealment** | **Participants blinded** | **Care providers blinded** | **Outcome assessors blinded** | **Incomplete outcome data** | **Selective outcome reporting** |
| --- | --- | --- | --- | --- | --- | --- | --- |
| Gillespie BM et al. (2015) | LOW | LOW | LOW | LOW | LOW | LOW | LOW |
| Howell RD (2011) | UNCLEAR | LOW | LOW | LOW | HIGH | UNCLEAR | LOW |
| Masden D et al. (2012) | LOW | LOW | LOW | LOW | HIGH | LOW | LOW |
| Stannard JP et al. (2006) | LOW | UNCLEAR | LOW | LOW | HIGH | UNCLEAR | LOW |
| Stannard JP et al. (2012) | LOW | UNCLEAR | LOW | LOW | HIGH | UNCLEAR | LOW |

Risk of bias observational studies

|  | **Selection** | | | | **Comparibility** | **Outcome** | | |
| --- | --- | --- | --- | --- | --- | --- | --- | --- |
| **Author, year** | **Representativeness of cohort** | **Selection of non-exposed cohort** | **Ascertainment of exposure** | **Demonstration that outcome of interest was not present at start** | **Comparibility of cohorts** | **Assessment of outcome** | **Follow-up long enough** | **Adequacy of follow-up of cohorts** |
| Adogwa O et al (2014) | * | * | * | * | ** | * | * | * |
| Blackham AU et al (2013) | * |  | * | * | * | * | * | * |
| Bonds AM et al. (2013) | * |  | * | * | ** | * | * | * |
| Chadi SA et al (2014) | * | * | * | * | ** | * | * | * |
| Condé-Green A et al. (2013) | * | * | * | * | ** | * | * | * |
| Gassman A et al. (201) | * |  | * | * | * | * | * |  |
| Grauhan O et al. (2013) | * | * | * | * | ** | * | * | * |
| Grauhan O et al. (2014) | * | * | * | * | ** | * | * | * |
| Matatov T et al. (2013) | * | * | * | * | ** | * | * |  |
| Pellino G et al. (2014) | * | * | * | * | ** | * | * |  |
| Pauli EM et al. (2013) | * |  | * | * | * | * | * |  |
| Reddix RN et al. (2010) | * |  | * | * | * | * | * |  |
| Selvaggi et al. (2014) | * |  | * |  | ** | * | * | * |
| Soares KC et al. (2015) | * | * | * | * | * | * | * |  |

* A study can be awarded a maximum of one star for each numbered item within the Selection and Outcome categories.

A maximum of two stars can be given for Comparibility

**Appendix B. Funnel plots (left RCTs, right observational studies)**


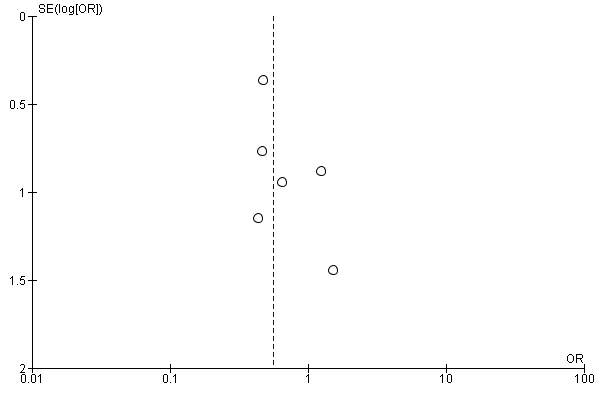


**Appendix C. Stratification by type of surgery (all RCTs)**

**Stratification by type of surgery (all observational)**


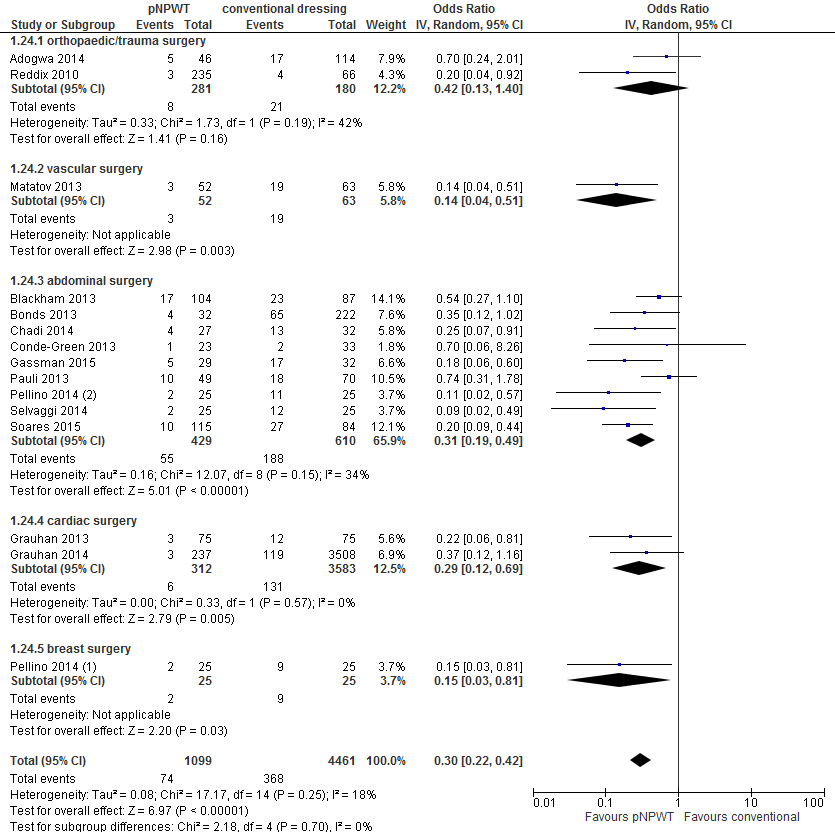

Supplement: Supplemental Digital Content [file medi-95-e4673-s001.doc]
